# The role of microRNAs in axon regeneration after peripheral nerve injury: a bibliometric analysis

**DOI:** 10.3389/fneur.2024.1348048

**Published:** 2024-03-06

**Authors:** Kutiluke Shoukeer, Shalayiding Aierxiding, Aikebaierjiang Aisaiti, Abuduwupuer Haibier, Chunhua Liu, Zhiwei Jia, Abudunaibi Aili, Li Shu, Kan Jiang, Aikeremujiang Muheremu

**Affiliations:** ^1^Department of Orthopedics, Sixth Affiliated Hospital of Xinjiang Medical University, Ürümqi, China; ^2^Research Department of Beijing Darwin Cell Biotechnology Co., Ltd, Beijing, China; ^3^Department of Orthopedics, Dongzhimen Hospital, Beijing University of Chinese Medicine, Beijing, China

**Keywords:** peripheral nerve injury, microRNAs, axonal regeneration, bibliomeric analysis, visualization

## Abstract

**Objective:**

This study analyzed the current research hotspots and future development trends of the therapeutic effects of microRNA on PNI axonal regeneration through bibliometric methods. Moreover, the current advantages and disadvantages of this field as well as future development prospects are discussed in depth.

**Methods:**

CiteSpace V and VOSviewer were used as bibliometric tools to complete the analysis of the research focus and direction of the published articles. To supplement, sort out, and summarize, we analyzed the research status of the study on the application of microRNAs for axonal regeneration after peripheral nerve injury from 2013 to 2023.

**Results:**

A total of 207 publications were retrieved from the Web of Science database. After exclusion and screening, a final selection of 174 articles that met the research criteria. These 174 articles were authored by a total of 846 individuals, representing 24 countries and 199 institutions. Additionally, this study presents information on the annual publication output, country distribution, top 5 contributing authors, top 5 most cited articles, and top 10 contributing institutions.

**Conclusion:**

As one of the hottest topics today, microRNAs have become the current research hotspot in neural inflammation, neural cell repair and regeneration, neural protection, and functional recovery. With more investment in research in this field, more high-quality articles will be published in both domestic and international outstanding journals, which will bring a new era for the treatment of peripheral nerve injury.

## 1 Introduction

Peripheral Nerve Injury (PNI) is a common clinical condition that is associated with sensory and motor impairments, chronic pain, and functional disabilities in the neural innervation region caused by traumatic and non-traumatic factors (1, 2). Relevant studies have shown that the annual incidence rate of PNI worldwide is approximately 13/100,000–23/100,000 (3–5). Peripheral nerve injuries not only result in severe limb functional impairments but may also lead to irreversible lifelong disabilities (6). Although the peripheral nervous system possesses some self-repair and regenerative abilities, the ultimate recovery outcomes often fall short of expectations (7). Currently, surgical treatment remains the preferred method for treating PNI, including procedures such as autologous nerve transplantation and tension-free nerve repair (8). However, long-term follow-up studies have found that some patients still experience persistent motor and sensory impairments without significant improvement or recovery (8). In recent years, various methods such as traditional Chinese medicine treatment (9), electrical stimulation therapy (10), and cell therapy (11) have been attempted for PNI, yet the medical community has yet to establish a universally acknowledged effective drug or approach for treating this disorder.

The peripheral nervous system is primarily composed of neurons and axons, with the axons being enveloped by myelin sheaths, which consist of Schwann cells, and the self-repair of injured nerves relies on axonal regeneration. After PNI, the regenerative capacity of axons is regulated by various factors, and microRNA (miRNA)-a non-coding single-stranded RNA, plays an important role in regulating the expression of post-transcriptional genes (12). It has been proven that miRNAs play a significant role in the pathogenesis of many diseases, including cancer, cardiovascular diseases, allergic diseases, autoimmune diseases, etc. (13–15). With the continuous development of medical and molecular biology technologies, an increasing number of scholars and institutions have devoted their efforts to the research on miRNA and related mechanisms, resulting in the expanding family of miRNA members. A total of 28 types of miRNAs have been discovered so far, and some miRNAs (such as miR-21 and miR-222) are upregulated after peripheral nerve injury, playing a role in promoting axonal growth and regeneration. They may achieve this by regulating the expression of genes including growth factors, cell adhesion molecules, and axonal guidance molecules, thus promoting axonal regeneration and repair (16, 17). Articles published in high-impact factor journals domestically and internationally have demonstrated the enormous potential of microRNA in peripheral nerve injury repair. In-depth research on the mechanisms of miRNAs in the process of neural regeneration will contribute to the development of treatment strategies targeting miRNAs, thereby promoting the recovery and repair of nerve injuries (18).

This study utilizes bibliometric analysis methods to visually analyze articles collected from the Web of Science database in terms of countries, authors, institutions, journals, articles, keywords, etc. It aims to explore the current hot topics and future development trends in this field, providing data and theoretical support for future research.

## 2 Data and methods

### 2.1 Data collection and retrieval

#### 2.1.1 Retriever

The screening and collection of all articles was conducted by a single scholar to minimize the impact of database updates and the potential variations caused by subjective selection. This meticulous process was successfully concluded on July 5, 2023.

#### 2.1.2 Search database and time limit

Web of Science (WoS), as one of the most common academic databases, includes over 12,000 academic journals (18). Therefore, this study utilizes the Web of Science as the data source for article selection and collection, with the retrieval date range set from January 1, 2013, to June 1, 2023.

#### 2.1.3 Search terms

Perform data retrieval on the Web of Science Core Collection in advanced search mode by entering the subject terms, such as: “microRNAs” and “axonal regeneration” or “microRNAs” and “peripheral nerve injury”; Using the search query “(TS = (microRNAs)) AND TS = (axonal regeneration) OR (TS = (microRNAs)) AND (TS = (peripheral nerve injury)).” Once the articles have been filtered, select the option to export them as a plain text file, including both full records and references. Then exported data should be analyzed using visualization analysis software such as CiteSpace 6.4.2R and VOSviewer 1.6.19 for further research.

**Table:** 

#1 subject term: microRNAs;
#2 free word: miRNA OR microRNA;
#3 #2 OR #1
#4 subject term: axonal regeneration;
#5 subject term: peripheral nerve injury;
#6 #3 AND #4 AND #5

#### 2.1.4 Inclusion criteria and exclusion criteria

Inclusion criteria: Relevant articles and reviews from the WOS database on the role of microRNAs in axonal regeneration following peripheral nerve injury were published between January 1st, 2013, and June 1st, 2023.

Exclusion criteria: (1) Duplicate literature; (2) Notifications, comments, translations, conference proceedings, abstracts, newspapers, patents, news, lectures, autobiographies, and graduation theses, among others.

### 2.2 Data analysis

This study utilized visualization analysis software CiteSpace6.4.2R, VOSviewer1.6.19, and Microsoft Excel 2019 for visualization processing. VOSviewer is a commonly used bibliometric tool developed and released by Nees Jan van Eck and Ludo Waltman in 2015. This software specializes in the visual analysis of bibliometric networks, where a group of related nodes can be allocated into distinct groups and differentiated by different colors, with nodes that have similar or close colors indicating higher relevance. CiteSpace is a software developed by Dr. Chaomei Chen and his team in 2004, which can used for visualizing and analyzing scientific literature. The software can help researchers analyze citation networks, keyword networks, and author collaboration networks, among other types of information, making it more convenient for researchers to understand the forefront of research fields, hotspots, and development trends. This study employed both software packages to generate graphical representations from different aspects, including annual publication distribution, country and region distribution, institutional distribution, popular authors, journals, citation and reference frequencies, and more, to comprehensively analyze and study the knowledge map. Keyword cluster maps and burst word maps were also generated for a more in-depth analysis.

## 3 Results

**Figure 1:**
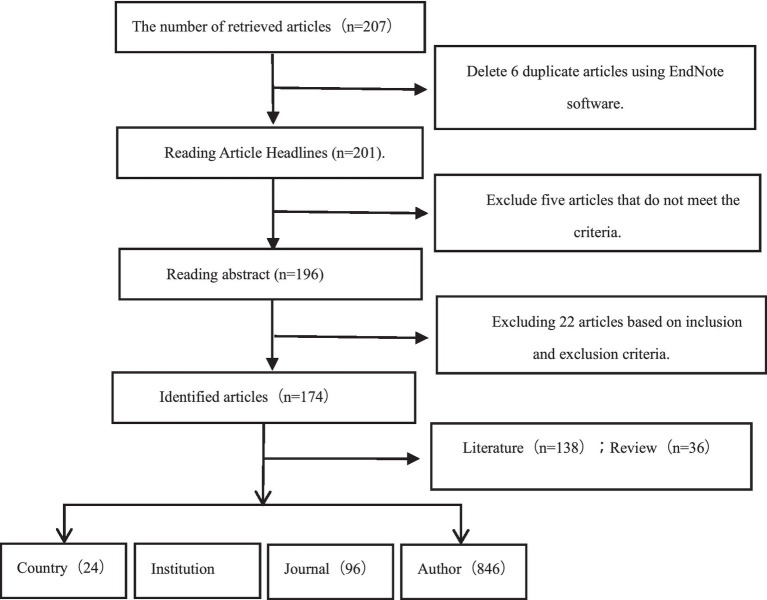
Flow chart of literature search and screening.

total of 207 articles were retrieved from the Web of Science database, after thorough elimination and screening, only 174 articles, that were relevant to the study were included. These 174 articles, which involved 846 authors, from 24 countries and 199 institutions and were published in 96 journals. In total, 8,824 references were cited from 1,493 journals (Figure 1).

### 3.1 Global publication trends

**Figure 2:**
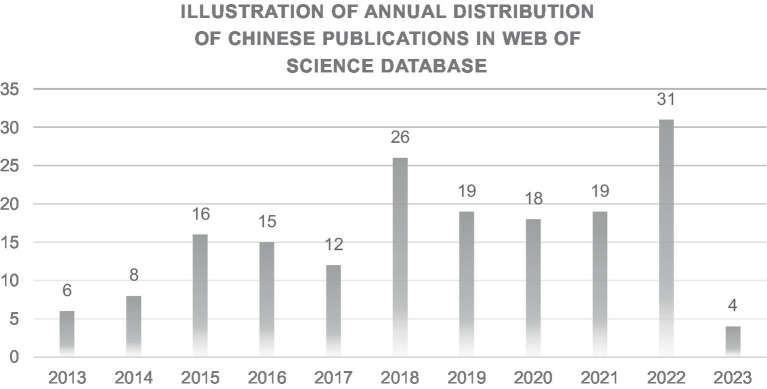
Schematic diagram of the annual distribution of literature publications in the Web of Science database.

The volume of published articles can demonstrate the current global trends and cutting-edge hotspots in a particular field (19). Figure 2 shows the global publication volume of articles related to the therapeutic effects of microRNA in axonal regeneration after PNI, collected from the Web of Science database over the past decade. It is not difficult to see that there were two peaks in the publication of articles in this field in 2019 and 2022. From 2015 to 2022, the number of articles published per year remained above 10, especially in 2022, when a total of 31 articles were published, the highest in the history of article publications so far. Although the annual distribution of article publications fluctuated slightly, the overall trend was that an increasing number of articles were published each year. Since the collection of research data ended on June 1, 2023, there are currently only 4 articles published in 2023. However, based on the average number of published articles in these years, there is still a good chance of exceeding 10 publications by the end of this year.

### 3.2 Country distribution

**Figure 3:**
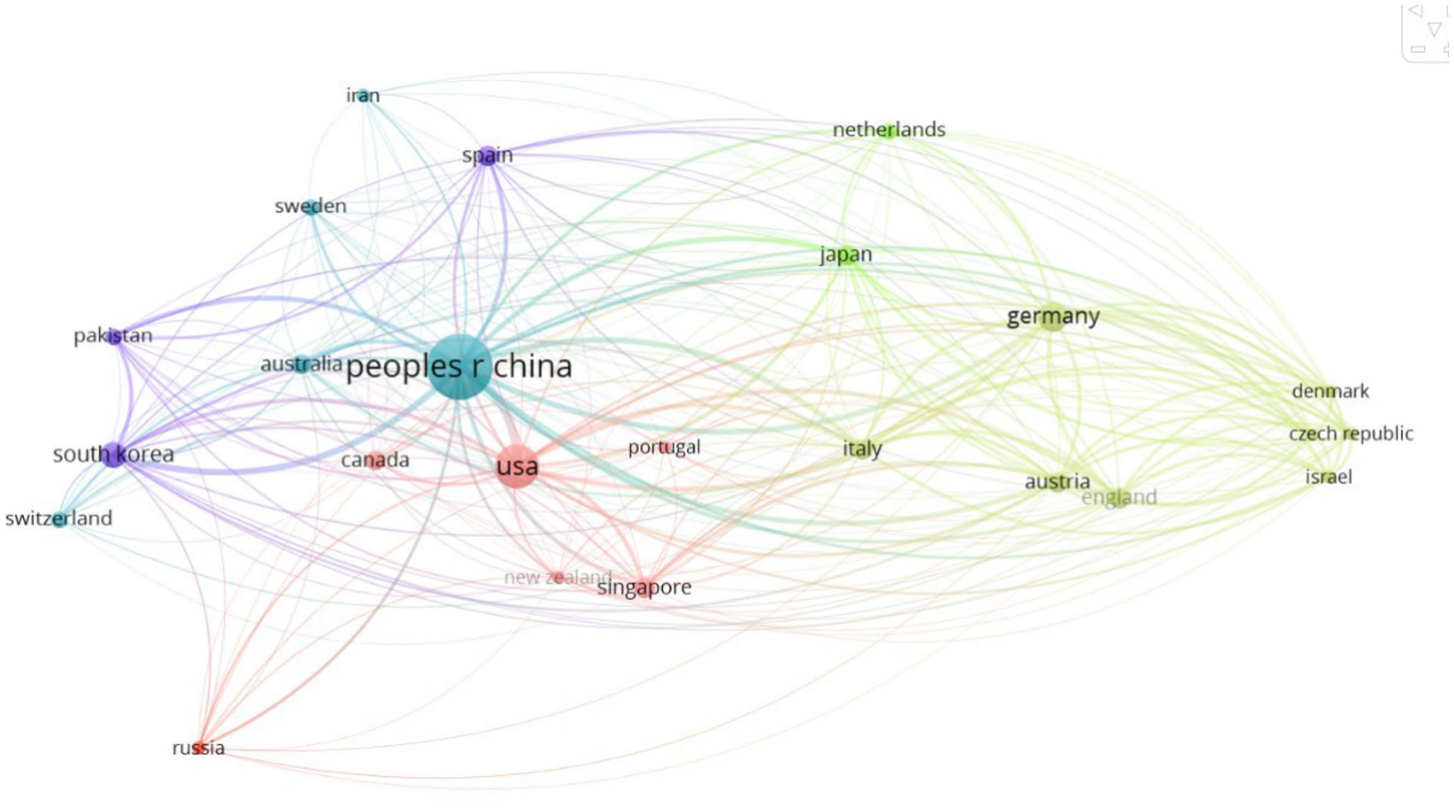
Distribution map of national and regional publications.

**Figure 4:**
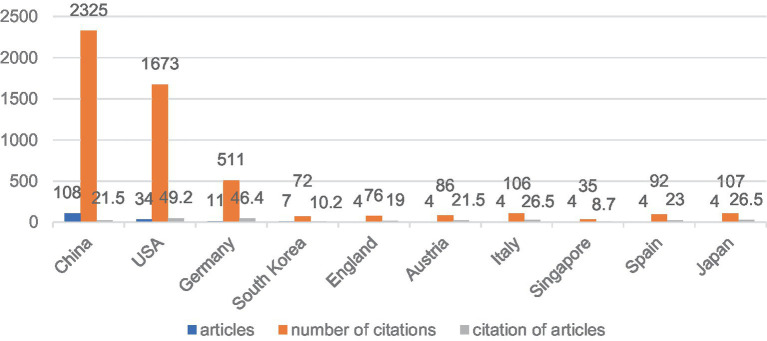
Distribution map of articles, number of citations, and citation of articles.

In terms of the number of articles published, China ranks first with 108 articles, while the United States ranks second with 34 articles (Figures 3, 4), and these two countries’ articles account for 81.6% of the total, indicating that China and the United States are in a dominant position in this field. In particular, China almost occupies half of the global article output, which highlights its leading role in the treatment of axonal regeneration after PNI using miRNA in the world. From the analysis of the number of citations, China and the United States still occupy the top two positions, with 2,352 and 1,673 citations, respectively, (Figure 4). However, the average number of citations per article is 49.2 and 46.4 for the United States and Germany, respectively, ranking first and second, while China ranks fifth with 21.5 citations, tied with Austria. This also reflects that although China ranks high in the total number of articles published, there is still a certain gap in the quality of the articles compared to developed countries in Europe and America.

### 3.3 Institution distribution

**Figure 5:**
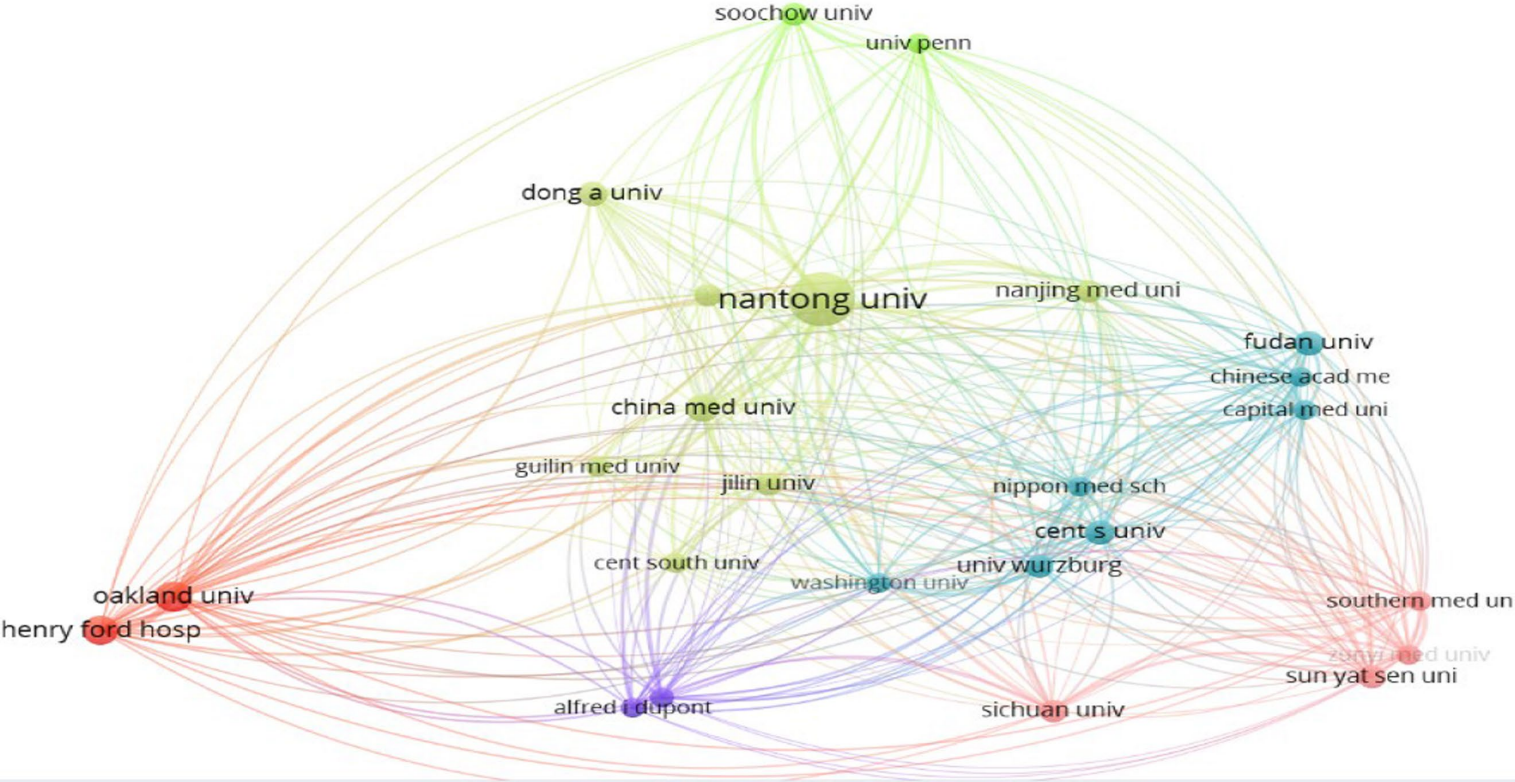
Study the cooperative network of relevant institutions.

A total of 199 institutions globally have contributed to the research field of the therapeutic effects of microRNA on axon regeneration after peripheral nerve injury. Among the top 10 institutions in terms of publication volume, 7 of them are from China, with Nantong University ranking first in publication volume with 31 articles and 734 citations. Although the University of Würzburg has only published 4 articles, its citation frequency of 81.2 times is significantly higher than that of other institutions, indicating the high academic recognition of the quality of its publications worldwide. Among Chinese institutions, Jilin University has the highest citation frequency of 54.5 times, ranking top among domestic universities in terms of article level and quality. Using Citespace software for cluster analysis of these institutions (Figure 5), the size of the nodes reflects the number of articles published, with larger nodes indicating a greater number of publications. The thicker the lines between each node, the more frequent the collaboration between the two institutions represented by the nodes. Analysis shows that domestic institutions tend to collaborate more frequently with other domestic institutions, with thinner lines and fewer publications in collaboration with foreign institutions” (Table 1).

**Table 1:** Top 10 institutions in the number of publications.

No.	Institution	Articles	Citation	Citation of articles
1	Nantong University	31	734	23.7
2	University of Auckland	8	198	24.7
3	Henry Ford Cancer Center	7	180	25.7
4	China Medical University	6	111	18.5
5	Nanjing Medical University	4	116	29.0
6	Soochow University	4	51	12.8
7	Jilin University	4	218	54.5
8	Sichuan University	4	91	22.7
9	Sun Yat-sen University	4	24	6.0
10	University of Würzburg	4	325	81.2

### 3.4 Author distribution

From 2003 to 2023, 846 authors worldwide have published relevant articles. Among the top five authors in terms of publication volume, four are from the Key Laboratory of Neuroregeneration at Nantong University. Professor Yi Sheng ranks first with 14 articles. In contrast, Academician Xiao Song Gu, the director of the Key Laboratory of Neuroregeneration at Nantong University, ranks first in terms of average citations per article with 49.5 (Table 2).

**Table 2:** Top five authors in terms of publications.

No.	Author	Articles	Citations	Average citations
1	Yi sheng	14	341	24.3
2	Xiao song Gu	10	495	49.5
3	Xing hui Wang	8	236	29.5
4	Tian mei Qian	8	192	24.0
5	Shi Ying Li	7	313	44.7

### 3.5 Journal distribution

**Figure 6:**
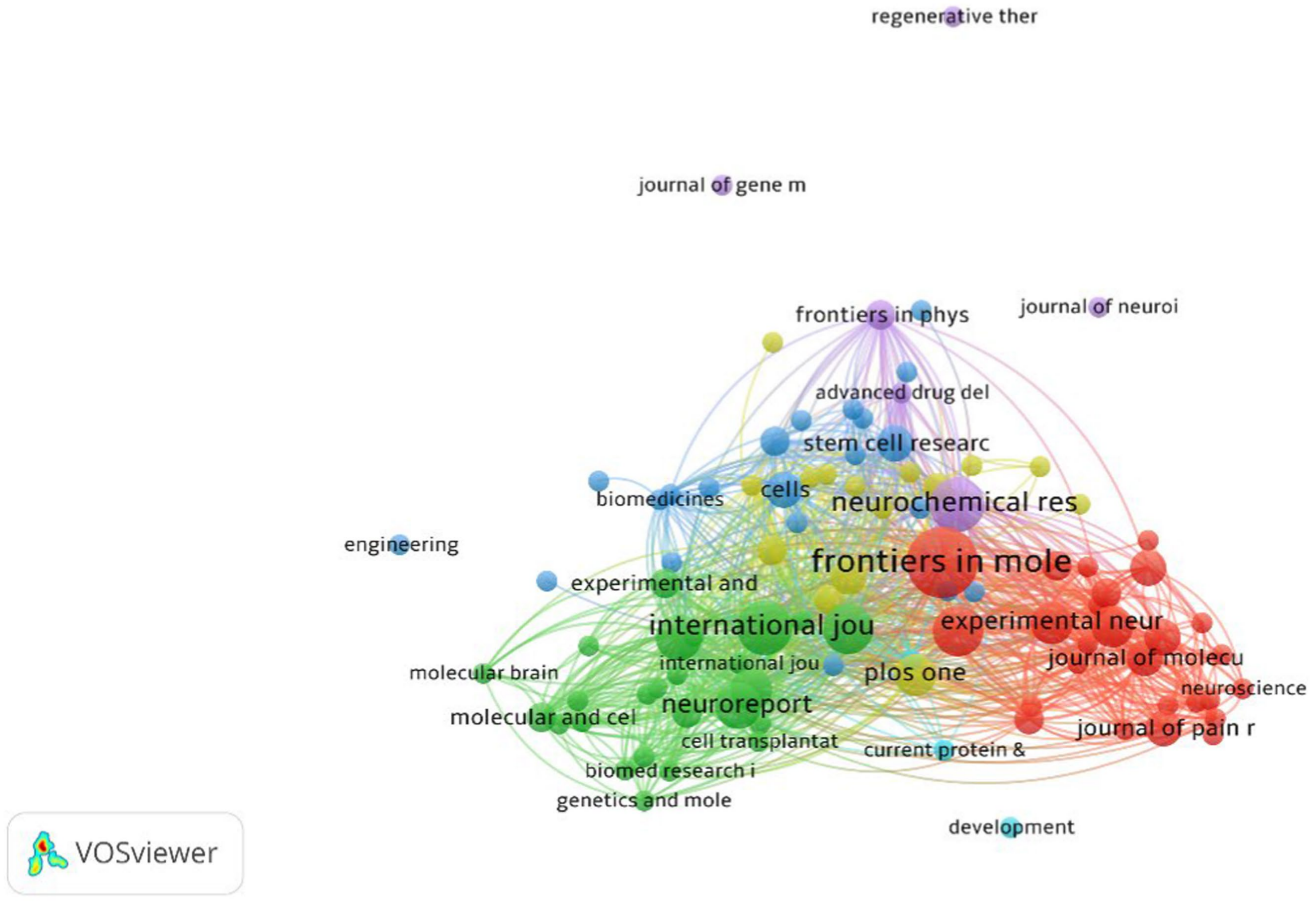
Research on the cooperative network of related journals. The circular nodes in the figure represent journals, with node size indicating the proportion of published articles. Larger nodes indicate a higher number of published articles. The connections between nodes represent the relationships between journals, with thicker or more numerous connections indicating stronger relationships.

Table 3 and Figure 6 illustrate the distribution of related academic journals publishing articles on the therapeutic effects of microRNAs in axon regeneration after PNI. The journal “Frontiers in Molecular Neuroscience” published the highest number of articles at a total of 12. Although both the “International Journal of Molecular Sciences” and the “Neurochemical Research” published the same number of articles, at 7, the latter received the most citations and had the highest average citations per article, with 230 and 32.8, respectively. The top five journals in terms of impact factor all exceeded 4.0, with an average of 5.19. “Neural Regeneration Research” had the highest impact factor, at 6.05.

**Table 3:** Top five journals in publication volume.

No.	Journal	Articles	Citation	Average citations	IF (2022)
1	Frontiers in Molecular Neuroscience	12	221	18.4	4.8
2	International journal of molecular sciences	7	56	8.0	5.6
3	Neurochemical Research	7	230	32.8	4.4
4	Molecular Neurobiology	6	138	23	5.1
5	Neural Regeneration Research	5	66	13.2	6.05

### 3.6 Article analysis

The number of citations can reflect the influence of an article in the target field (20). Table 4 lists the top 5 most cited articles worldwide, with Professor Shi Ying Li’s article “Let-7 microRNAs Regenerate Peripheral Nerve Regeneration by Targeting Nerve Growth Factor” published in the journal Molecular Therapy accumulating 34 citations. Meanwhile, Professor Bin Yu from the Key Laboratory of Neural Regeneration at Nantong University has 3 articles with citation counts of 27, 24, and 24 respectively, entering the top five rankings. This also demonstrates that Professor Yu has a wealth of knowledge and experience in the field. The five papers were published in five different journals, including Molecular Therapy, Journal of Cell Science, PLOS ONE, the Journal of Neuroscience, and Nucleic Acids Research, all of which are influential journals in the field of biomedical research. All of the papers focus on the role of microRNAs in neural injury and regeneration, indicating that microRNAs are a hot research topic in this field.

**Table 4:** Top five most popular cited paper.

No.	Title	Citation	Author	Journal
1	Let-7 microRNAs Regenerate Peripheral Nerve Regeneration by Targeting Nerve Growth Factor	34	Shi Ying Li	Molecular therapy
2	miR-221 and miR-222 promote Schwann cell proliferation and migration by targeting LASS2 after sciatic nerve injury	27	Bin YU	Journal of Cell Science
3	Profile of MicroRNAs following Rat Sciatic Nerve Injury by Deep Sequencing: Implication for Mechanisms of Nerve Regeneration	24	Bin YU	PIOS ONE
4	MicroRNAs modulate Schwann cell response to nerve injury by reinforcing transcriptional silencing dedifferentiation-related genes	25	Andreu Viader	The journal of neuroscience
5	miR-182 inhibits Schwann cell proliferation and migration by targeting FGF9 and NTM, respectively at an early stage following sciatic nerve injury	24	Bin YU	Nucleic acids research

### 3.7 Keyword analysis

**Figure 7:**
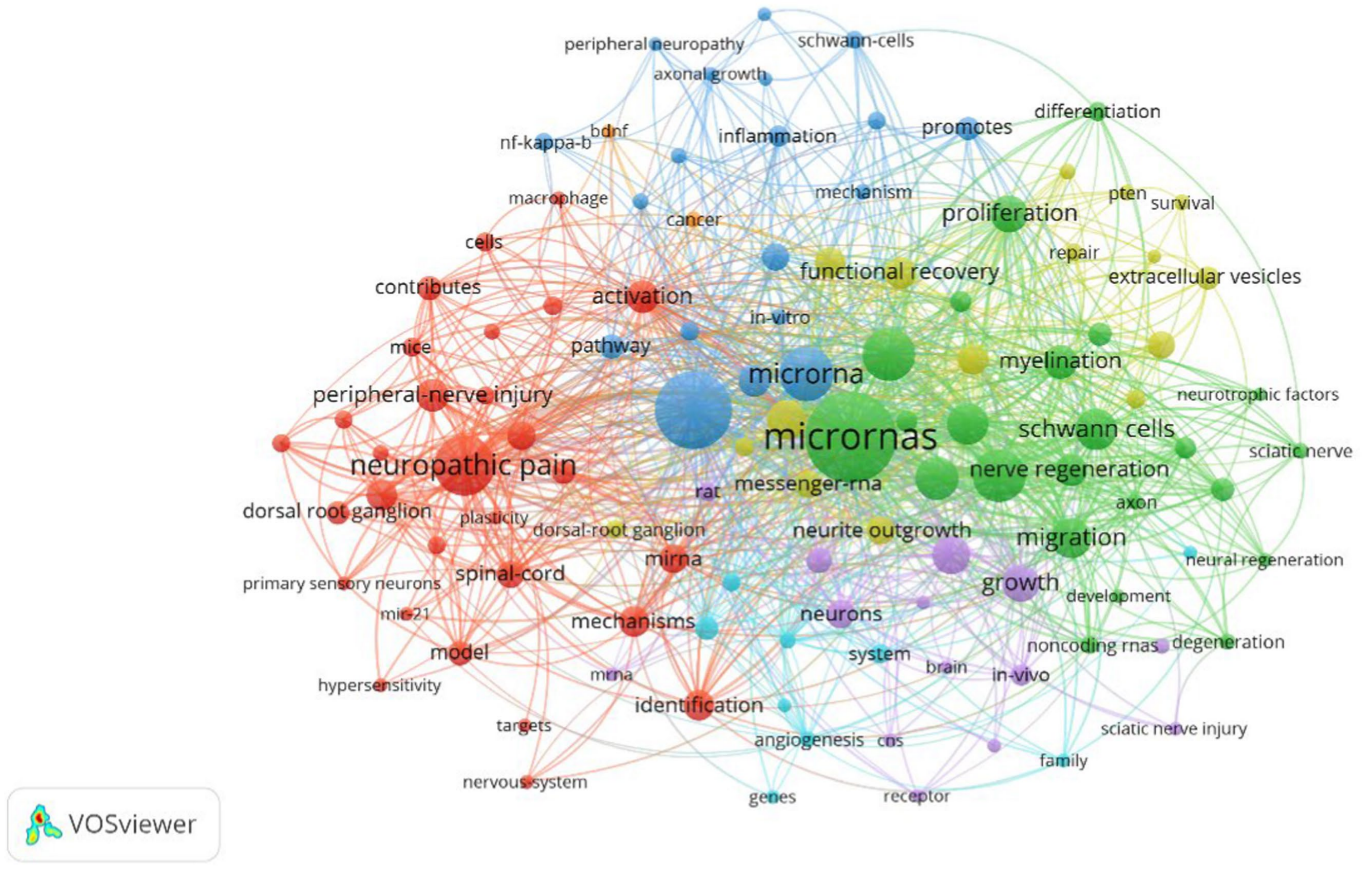
Keyword co-occurrence cluster map. In the figure, circular nodes represent journals, and node size represents the frequency of keywords. The larger the node, the higher the frequency. The color of the nodes represents the cluster of keywords. The connections between nodes represent the relationships between them. The more connections or thicker the lines, the stronger the relationship between the nodes.

**Figure 8:**
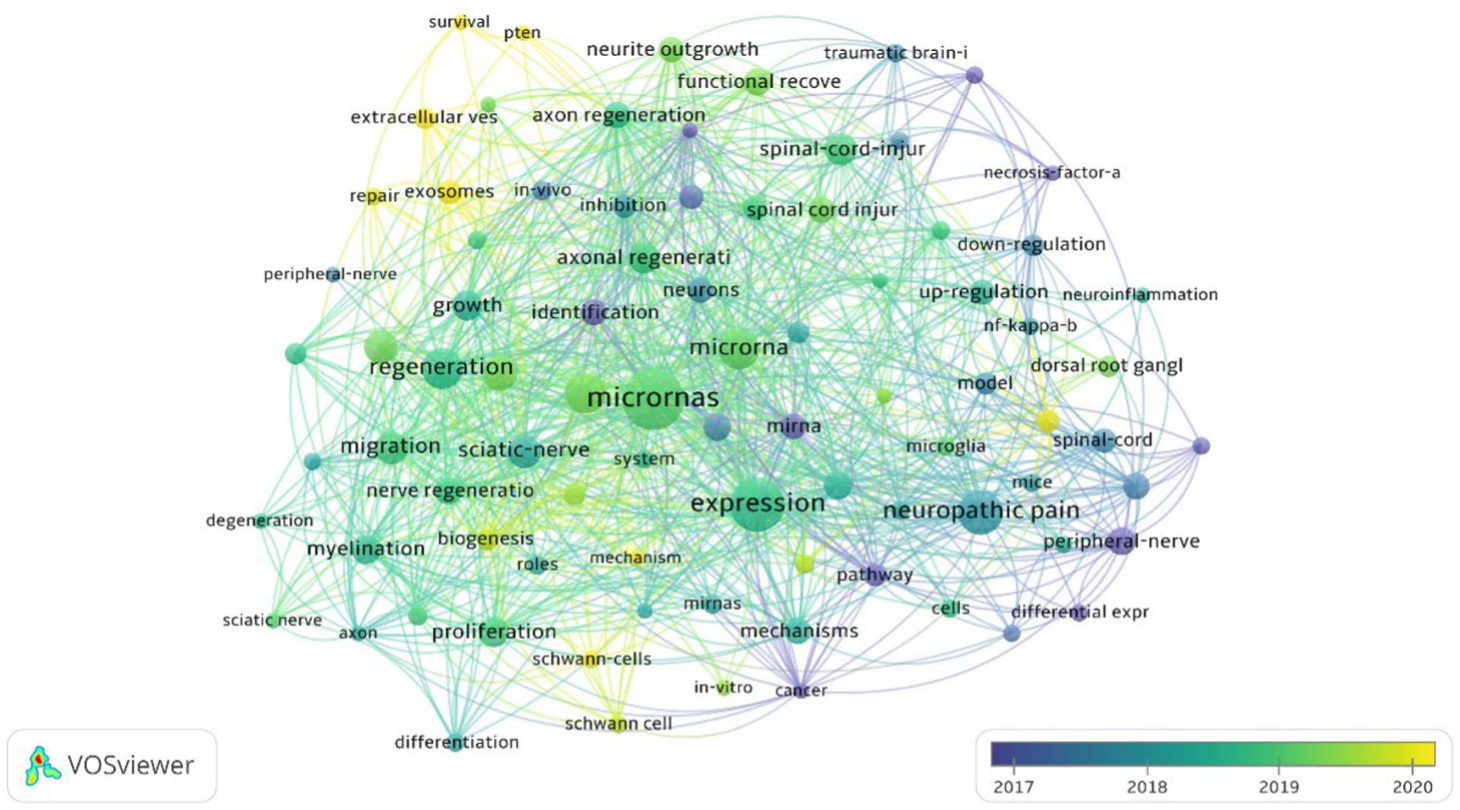
Keyword co-occurrence cluster map in 2013–2022 (time superposition). The circular nodes in the graph represent keywords, and each node's size represents the keyword's frequency. The larger the node, the higher the frequency. The color of each node represents the temporal frequency of the keyword, with lighter colors indicating a higher frequency. The lines connecting the nodes represent the strength of their relationship, with more or thicker lines indicating a stronger connection.

**Figure 9:**
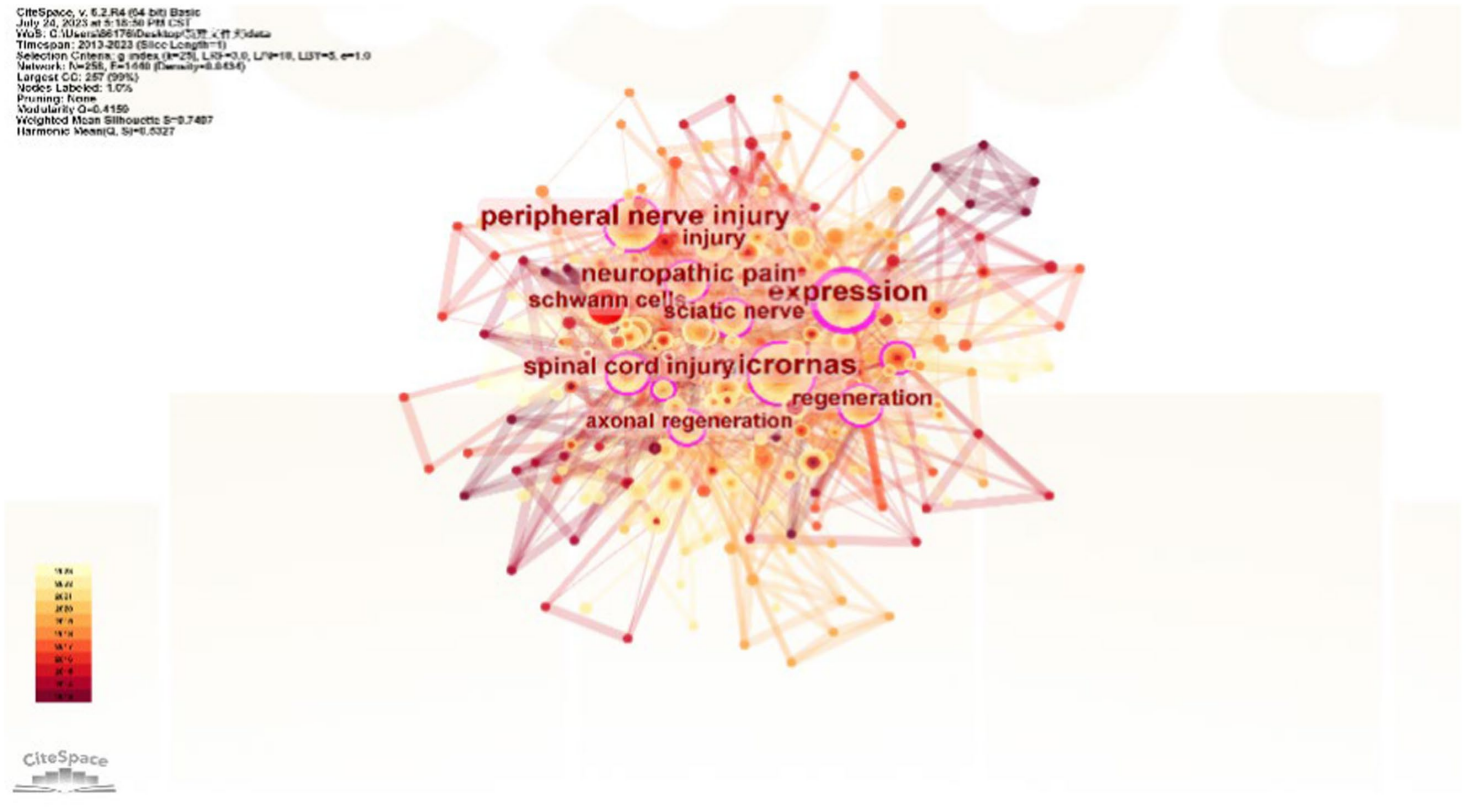
Keywords with frequency > 20.

The role of keywords in an article is significant, as they can more or less extract and reflect the core of an article. Among the 1,047 collected keywords, the top 10 are microRNAs, expression, neuropathic pain, miRNA, peripheral nerve injury, regeneration, sciatic-nerve, Schwann cell, migration, and proliferation (Table 5). MicroRNAs have appeared a total of 79 times and are the most frequently used keyword. Through VosViewer analysis (Figure 7), the 37 keywords that appeared more than 10 times were categorized into three clusters, and the size of each node reflects its frequency. MicroRNAs have become the most representative term in this field. Figure 8 reflects the relationship between keyword nodes and time. The closer the node color to yellow, the more recent the appearance of the keyword. It is not difficult to find that the majority of the keyword frequency distribution concentrates between 2016 and 2023. By analyzing the frequency of keywords, with CiteSpace visualization software, it is equally found that microRNAs are the most popular keyword (Figure 9). This also shows that this field is closely related to microRNAs in terms of development trends.

**Table 5:** Top 10 keywords in occurrence frequency.

No.	Keywords	Frequency of occurrence	Total connection strength
1	MicroRNAs	79	150
2	Expression	61	114
3	Neuropathic pain	41	61
4	miRNA	35	49
5	Peripheral nerve injury	34	84
6	Regeneration	34	73
7	Sciatic-nerve	23	76
8	Schwann cells	23	71
9	Migration	22	70
10	Proliferation	19	30

### 3.8 Analysis of outbreak keywords

**Figure 10:**
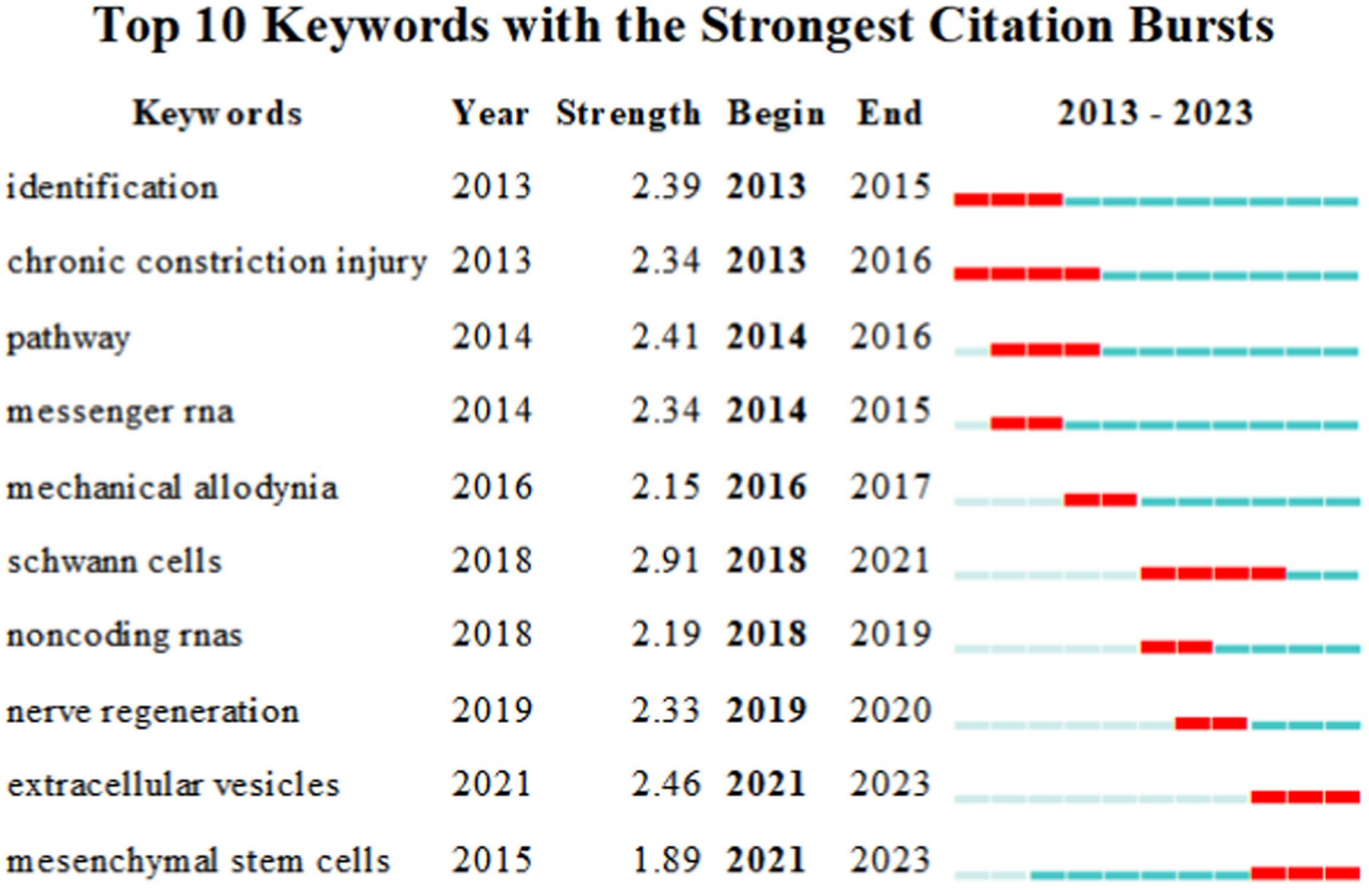
Keywords with frequency > 20.

The outbreak keywords phenomenon refers to the increase in the frequency of a certain keyword during a specific period. Figure 10 displays the top 10 burst word diagrams, where the keyword with the highest burst intensity is ‘Schwann cell’, which first appeared in 2018 and experienced a four-year burst. Schwann cells, as glial cells unique to the peripheral nervous system, possess remarkable plasticity and play a crucial role in axonal regeneration after PNI, as evidenced by a great deal of research (7, 20, 21). The most recent burst keyword is ‘mesenchymal stem cells’, which started to become the hotspot in 2021 and continued until 2023. Mesenchymal stem cells are a type of cell with self-replication ability and differentiation potential. Andrea Lavorato et al.’s study (22) elucidated the application prospects of this cell in peripheral nerve regeneration. Chen et al.’s 2019 research (23) confirmed that Schwann cell-like cells differentiated from mesenchymal stem cells could promote myelin sheath formation, and miRNA-24 overexpressed in SCLC contributed to the therapeutic effect of sciatic nerve injury in SCLC. A recent study has shown that miRNAs can regulate the proliferation, differentiation, and immunoregulatory functions of MSCs (24). By regulating the expression levels of miRNAs, the biological characteristics of MSCs can be affected, further influencing their potential for tissue repair and regeneration (25). Additionally, MSCs themselves can release extracellular vesicles rich in miRNAs, and miRNAs delivered by these vesicles can affect the gene expression and function of surrounding cells (26).

## 4 Discussion

### 4.1 Bibliometrics

Bibliometrics, as a branch of information science, plays an increasingly important role in the field of medicine. It not only provides researchers with visual and simplified knowledge structures, but also reflects the overall trends, hotspots, and directions of the research content (27, 28). The collected literature data, after being visualized and mapped using CiteSpace 6.4.2R and VOSviewer 1.6.19, can greatly assist authors in analysis and research. Web of Science is a data services platform provided by Clarivate Analytics, as the largest and most comprehensive academic resource library in the world, covering biomedical, engineering, natural science, social science, humanities, and arts, with more than 8,700 core journals, regarded as the data source platform by this study (25). It also includes information on cited references, authors, sources, and publication years. Furthermore, it offers powerful functions such as quick search, advanced search, and citation search. It not only provides information on the latest articles but also on high-quality journals from the past. Therefore, using the Web of Science Core Collection as the data source is most appropriate for this study (29, 30).

### 4.2 Global research status

#### 4.2.1 National literature publication and quality assessment

Analysis of the relevant literature in the past decade reveals that the average annual publication of articles related to the therapeutic role of microRNAs in axonal regeneration after peripheral nerve injury was 17.4 from 2003 to the present. In 2022, there was a small peak in this field with a total of 31 publications for the year. Except for 2018 and the remaining years of the study period, the number of publications was below 20. Although the number of publications has been gradually increasing, it is still relatively low compared to other fields. Regarding article types, nearly 78% of the articles were research articles, reflecting the researchers’ focus on original research rather than review articles.

The number of articles published in a certain research field can to some extent evaluate the scientific research level of a country and institution in that field (31). Looking at the countries involved, China leads in the number of publications among all countries, surpassing the second-ranked United States by 74 publications. Moreover, only China, the United States, and Germany had double-digit publication numbers. The number of citations and the average citations per article reflect the quality of an article, and high-impact factors and high-quality articles often receive more citations. In this ranking, the United States and Germany ranked first and second with 49.2 and 46.4 citations, respectively, far ahead of other countries, while China ranked sixth. Developed countries in Europe and America attach more importance to the quality of articles than China does.

As a populous country, China has a large population and a high number of patients. According to a study, the annual number of new PNI patients in China is 600,000 to 900,000 (32). With the gradual aging of society and the unequal development of medical conditions, improving patient prognosis and reducing complications have become difficult challenges for medical researchers in our country. While improving medical standards, the development of research quality should not be neglected. Only when diagnostic and treatment techniques and research levels progress together can we fundamentally solve the difficulties and problems in medical practice and narrow the gap between us and developed countries in Europe and America.

#### 4.2.2 Evaluation of the quantity and quality of institutional publications

In the distribution of authors and institutions publishing articles in the field of microRNAs in axonal regeneration after PNI, the Neuroregeneration Key Laboratory of Nantong University has made the largest contribution. Among the top five authors in terms of publication volume, four are from this institution, and Professor Xiao Song Gu (33), as the leader of this institution, has made great contributions to this field. Professor Yi Sheng also ranked first in terms of the number of articles published in this field, with 14 publications. His research on the promotion of remyelination of Schwann cells after PNI by miR-30c in 2017 received a good response in this field. He found that the functional study of miRNA-30c helps to better understand the regulatory role of miRNA in peripheral nerve regeneration and may provide new therapeutic methods for peripheral nerve injuries (34). Another professor from Nantong University, Professor Yu Bin, received a high number of citations for all three of his articles. This also indicates that Professor Yu Bin has a solid knowledge base and a high-quality research level in this field. Regarding institutional distribution, Chinese institutions still occupy the largest proportion, with Nantong University ranking first in terms of publication volume, while the University of Würzburg from Germany ranks first in terms of average citations per article. This once again confirms the gap in the quality of research articles between China and developed foreign countries. Enhancing collaboration among different research institutions or teams is of great significance for future research, whether it be in basic scientific research or clinical trials.

### 4.3 Future research directions and development trends

MicroRNAs are undoubtedly a popular research direction in contemporary medical fields, and the co-occurrence of popular keywords further verifies this fact. Through analysis of the knowledge graph, we can see that all the keywords included in the research are divided into three different clusters, and microRNAs have a high frequency in all three clusters. The red cluster mainly covers the expression pathway and neuropathic pain, the green cluster mainly includes peripheral nerves, Schwann cells, and the sciatic nerve, and the yellow and purple cluster involves axon regeneration, repair, and functional recovery. These clusters reflect the current hotspots and future development trends in this field. The red cluster represents the expression pathway-related field, the green cluster represents the neurological field, and the yellow cluster represents the neural repair and regeneration field. In addition, we can also observe that before 2016, early research was mainly focused on gene expression and neuropathic pain-related directions, while after 2016, the focus of research began to shift toward neural and injury repair directions. This further infers that future development trends will mainly focus on research in neural regeneration and functional recovery. Studying the role of different microRNAs in neural regeneration and gene expression regulation to reveal specific mechanisms and find effective diagnosis and treatment methods for PNIs has become a frontier hotspot and future research direction in this field.

The limitations of this study can be summarized as follows: firstly, the collected and screened article data are all collected from the Web of Science Core Database. Although this database is considered the most complete and authoritative database, data omissions are inevitable. Secondly, this study only included English-language articles, and high-quality articles from non-English classes were not included in the analysis, which may lead to selection bias. Last but not least, the Web of Science Core Database is updated in real-time, so the analysis results we obtained in this study have temporal limitations. However, existing research still provides useful insights and guidance for our research direction and design.

## 5 Conclusion

This article, through a bibliometric analysis of microRNA in the field of peripheral nerve injury treatment, systematically summarizes the research hotspots and current status in this area for the first time. As a cross-disciplinary subject between orthopedics and neurology, PNI has great development prospects. With microRNA becoming a hot topic in the medical field today, the study of its mechanism of action in PNI repair has become a breakthrough in improving patient prognostic quality, and the number of related publications has been increasing in recent years. China and the USA can be regarded as the largest contributor in this field. Through the analysis of articles, keywords, and burst words, we found that miRNA plays an important role in the process of axonal regeneration after PNI through expression regulation. The study of miRNA in areas such as neuroinflammation, neural cell repair and regeneration, neuroprotection, and functional recovery has become a current research hotspot. Future research directions will focus on exploring the regulatory mechanisms of miRNA in these areas and developing new treatment strategies and therapies to promote the repair and recovery of PNI. This will bring new hope and opportunities for us to better understand and treat nerve injuries. We believe that with the continuous increase in related research and articles in this field, the repair of PNI will usher in a new era. We aspire for our study to offer profound insights and serve as a valuable reference in propelling scientific advancements in related fields. Subsequent research endeavors and clinical practices will be better positioned to harness these scientific contributions, fostering innovation, and facilitating the application of microRNA in the treatment of peripheral nerve injuries.

## Data availability statement

The original contributions presented in the study are included in the article/supplementary material, further inquiries can be directed to the corresponding author.

## Author contributions

KS: Writing – original draft, Writing – review & editing. SA: Formal analysis, Writing – original draft. AiA: Funding acquisition, Writing – original draft. AH: Validation, Writing – review & editing. CL: Project administration, Writing – review & editing. ZJ: Supervision, Writing – review & editing. AbA: Visualization, Writing – review & editing. LS: Writing – review & editing. KJ: Writing – review & editing. AM: Writing – review & editing.
